# Supplementary material for: Geometric frustration in polygons of polariton condensates creating vortices of varying topological charge
Source: Nat Commun. 2021 Apr 9;12:2120. doi: 10.1038/s41467-021-22121-3 (PMC8035188; doi:10.1038/s41467-021-22121-3)
Supplement: Supplementary file 1 — Supplementary Information [file 41467_2021_22121_MOESM1_ESM.pdf]

# Geometric frustration in polygons of polariton condensates creating vortices of varying topological charge: Supplementary Information

Tamsin Cookson[1,2], Kirill Kalinin[1,3], Helgi Sigurdsson[1,2], Julian D. Töpfer[1,2], Sergey Alyatkin[1], Matteo Silva[2], Wolfgang Langbein[4], Natalia G. Berloff[1,3], and Pavlos G. Lagoudakis[1,2]

[1]Skolkovo Institute of Science and Technology, Skolkovo 143025, Russian Federation.

[2]Department of Physics and Astronomy, University of Southampton, Southampton, SO17 1BJ, United Kingdom.

[3]Department of Applied Mathematics and Theoretical Physics, University of Cambridge, Cambridge CB3 0WA, United Kingdom.

[4]School of Physics and Astronomy, Cardiff University, The Parade, Cardiff CF24 3AA, United Kingdom.

## I. 2D GROSS-PITAEVSKII SIMULATION PARAMETERS

The values for the parameters appearing in Equations (1) and (2) to simulate the two-dimensional driven-dissipative Gross-Pitaevskii equation are specified here. The polariton mass and lifetime are based on the sample properties: $m^* = 0.35$ meV ps$^2$ µm$^{-2}$ and $\gamma^{-1} = 5.5$ ps. We choose values of interaction strengths typical of GaAs based systems: $\alpha = 3.3$ µeV µm$^2$ and $g = 2\alpha$. The redistribution rate of reservoir excitons is taken here as comparable to the condensate decay rate $\Gamma^{-1} = 5$ ps and the damping parameter is chosen small $\Lambda = 0.05$. The final two parameters are found by fitting numerical results to experiment which gives $\hbar R = 33$ µeV µm$^{-2}$, and $G = 66$ µeV µm$^{-2}$. The $n$-th pump element (vertex) is written as a Gaussian profile $P_n(\mathbf{r}) = P_0 e^{-r_n^2/2w_{\text{RMS}}^2}$ where $r_n = \sqrt{(x-x_n)^2 + (y-y_n)^2}$, and $(x_n, y_n)$ denote the coordinates of the element. Here $P_0$ denotes the pump power density, and $w_{\text{RMS}} = 1.27$ µm the RMS width (corresponds to a 3 µm full-width-half-maximum), slightly larger than the incident light beam width in order to account for the small diffusion of excitons from the pump spots.

## II. EXPERIMENTAL NON-VORTEX CONFIGURATIONS

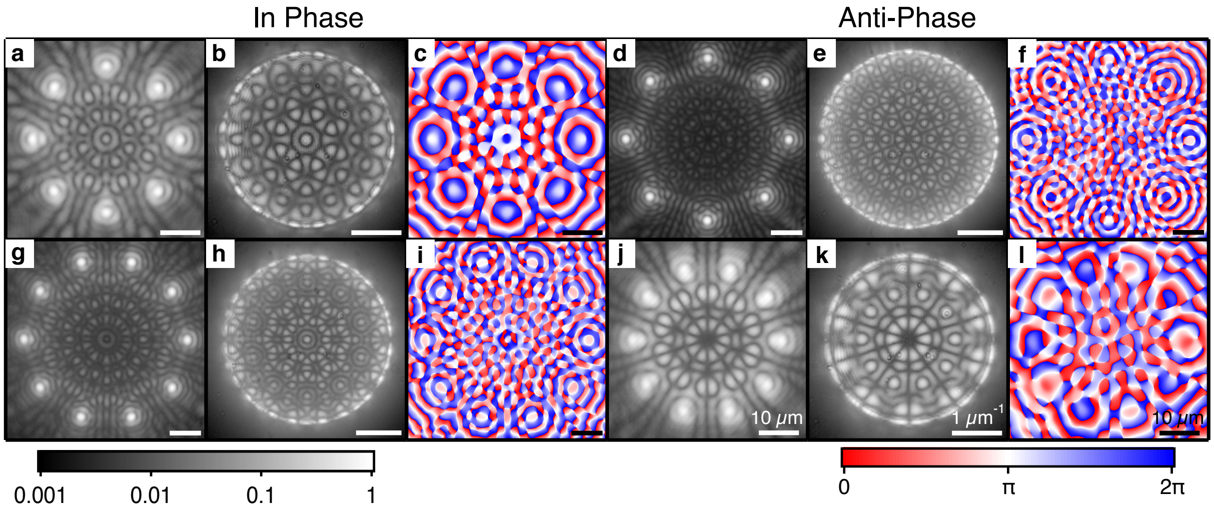


FIG. S1. **Even numbered polygons in in-phase and anti-phase configurations.** Real- (a,d), Fourier-space PL (b,e), and real-space phase (c,f) of an octagon condensate in in-phase and anti-phase configurations. (g-l) Same measurements for a decagon condensate. The phase is extracted from time-averaged interferograms with off-axis digital holography. Scale bars in real-space and the phase represents 10 μm, Fourier-space represent 1 $\mu m^{-1}$, as shown in the bottom right images.

Condensate octagon and decagon geometries are displayed in Fig. S1, synchronised in in-phase (left hand side) and anti-phase (right hand side) configuration with respect to nearest-neighbour condensates. The in-phase states in Figs. S1(a,g) show the real-space configuration with an odd number of fringes between nearest neighbour condensates (in this case three fringes). In real- (Figs. S1(a,g)) and Fourier-space (Figs. S1(b,h)) both display a bright spot at the centre indicating that all spots are in-phase. The phase in Figs. S1(c,i) reflect the clear pattern in the real-space and it can be seen that the spot centres are all at the same phase. Conversely, the anti-phase configurations have a dark spot at the centre and display clear radial nodal lines in real- Figs. S1(d,j) and Fourier-space Figs. S1(e,k). The corresponding polariton phase maps are shown in Figs. S1 (f,l), where neighbouring spots can be clearly observed in anti-phase.

Similarly, in the non-frustrated regime ($J > 0$) odd numbered pump polygons will form condensates in the in-phase configuration. The in-phase state has a bright fringe between adjacent condensates as well as the bright central fringe in both real- and Fourier-space

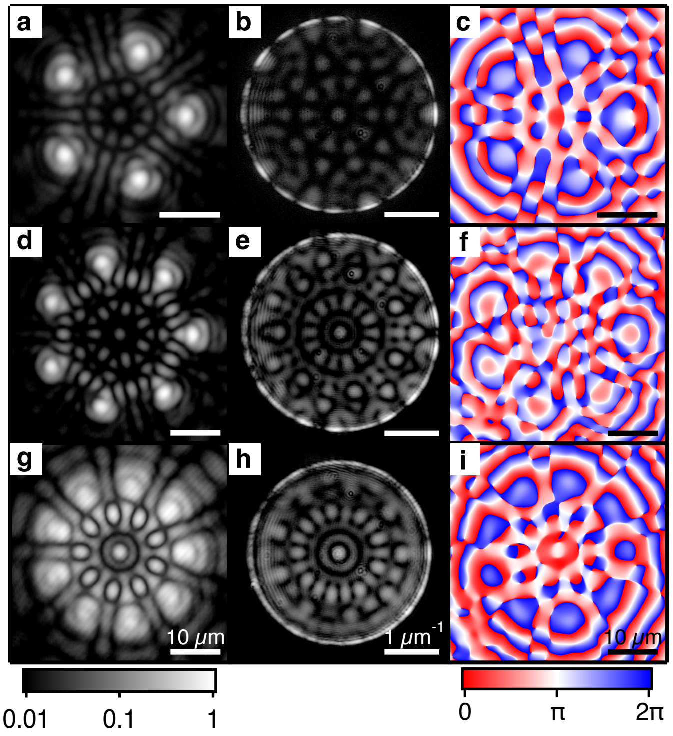


FIG. S2. **Odd numbered polygons in in-phase configuration.** Real- (a), Fourier-space PL (b), and real-space phase (c) of a pentagon in in-phase configuration. Same measurements are shown for heptagon (d-f) and nonagon condensates (g-i). The phase is extracted from time-averaged interferograms with off-axis digital holography. Scale bars in real-space and the phase represents 10 μm, Fourier-space represent 1 $\mu m^{-1}$, as labelled in the bottom row.

images, as shown in Figs. S2(a,b,d,e,g,h) for a pentagon, heptagon and nonagon, respectively. The corresponding polariton phase maps are shown in Figs. S2(c,f,i), where the neighbouring spots can be seen to be in-phase.

## III. RADIUS SCAN OF HEPTAGON

A scan of the radius of a heptagon was performed whilst integrating over multiple instances of the condensate as the real-space shows in Fig. S3. The system was found to be quite robust and stable over the course of the measurement. The system is initially in the "fifth" in-phase configuration at 19.81 μm corresponding to five bright fringes observed between neighbouring condensates and a bright notch at the centre. Upon increasing the radius, the system alters whilst it does retain five fringes between vertices, the centre now

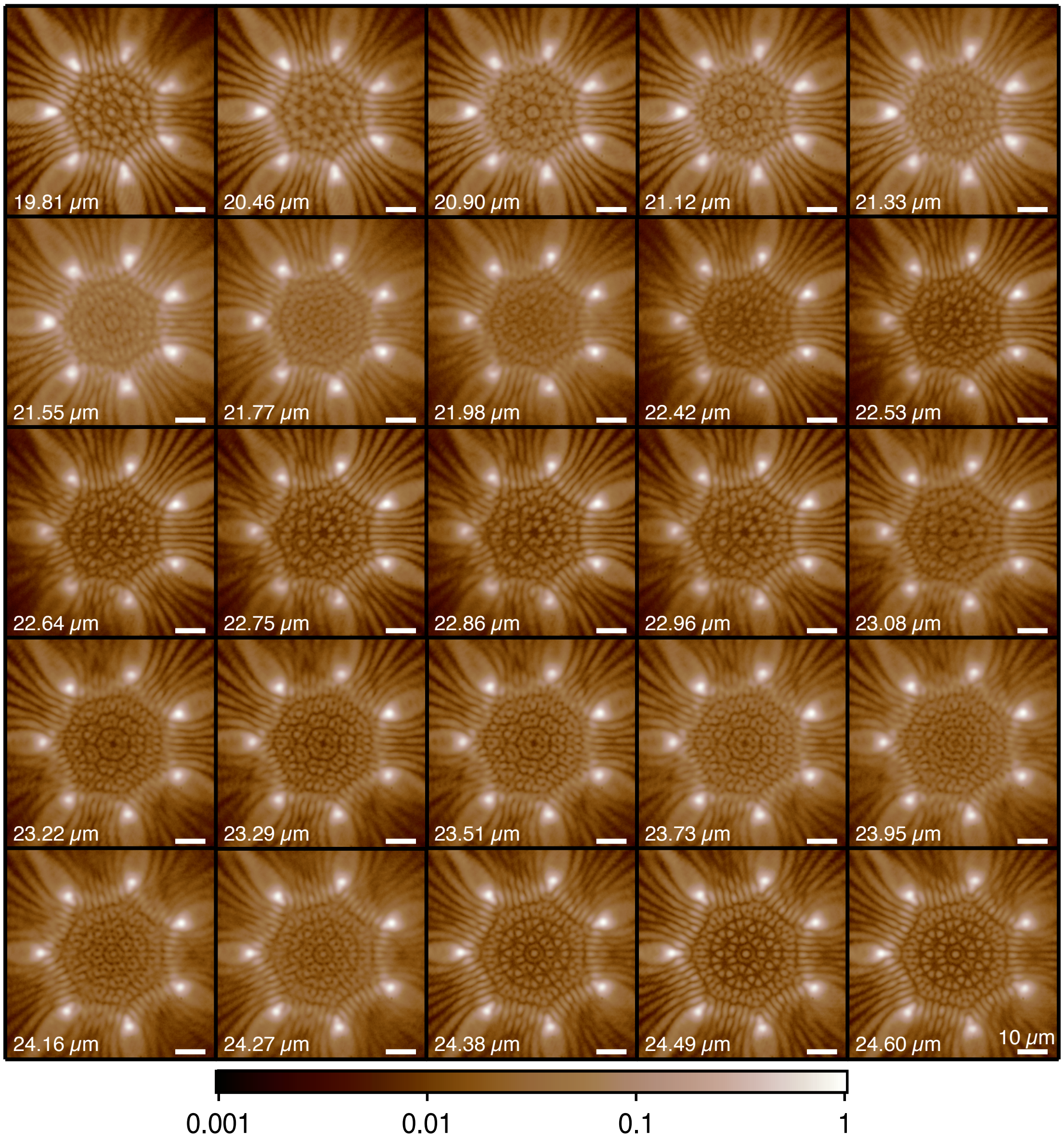


FIG. S3. **The radius scan of a heptagon.** Increasing the radius of a heptagon from ∼ 19.81 μm to ∼ 24.6 μm. The normalised real-space can be seen to go from in-phase with five fringes between neighbouring condensates at 19.81 μm; to the first vortex state at 21.12 μm; to the second vortex state at 23.29 μm; and back to in-phase at 24.38 μm, where seven fringes can be seen between neighbouring condensates. Between these states there are several fractured states. The third vortex state, which would contain a three-fold vortex with a full phase rotation of $6\pi$ was not observed in this run. All images are plotted on the same scale as defined by the scale bar in the bottom right.

has a small dark notch surrounded by a bright ring, most clearly seen at a radius of 21.12 μm corresponding to the first vortex state forming. Further increase of the radius caused the state to fracture one or more of the condensates similar to the vortex-antivortex state demonstrated in Fig. 5 (in the main text). Furthermore, at a radius of 22.53 μm, two of the condensates can be seen in the integrated real-space to be splitting (middle left-hand and bottom left-hand condensates), indicative that the frustration in the polygon has caused some of the condensates to split. A different number of fringes can be seen between different vertices (either five or six).

The next state to occur is seen at 23.29 μm with six fringes between vertices, where the real-space pattern has a heptagon at its centre surrounding a dark notch, (Fig. S3, fourth row, second image). The heptagon pattern in real-space comes from several instances of both $+\theta_{i,i+1}$ and $-\theta_{i,i+1}$ being time integrated causing a merging of the patterns and reduced visibility in the fringes. The heptagon then eventually returns to in-phase configuration at a radius of 24.38 μm with seven fringes between neighbouring condensates (Fig. S3, bottom row, third image).

## IV. SIMULATED VORTEX FORMATION STATISTICS

In Fig. S4(a) (red dot-dashed curve) we show the probability of a $|m| = 2$ vortex forming in a pentagon geometry by simulating Eq. 1 from stochastic initial conditions, averaged over 30 realisations (Monte-Carlo methods). The horizontal axis represents the short distance $d$ between neighbours (edge length) which is related to the polygons radius $R$ through the formula $d = 2R\sin(\pi/N)$. Between the regions of vortex formation, the probability of in-phase solutions (blue curve) forming becomes dominant as expected. The probability is estimated from the root-mean-square error of the condensate phase at each vertex,

$$\text{ERR}(\bar{\theta}) = \sqrt{\frac{1}{2N}\left[\sum_{n=1}^{N}(\cos(\bar{\theta}) - \cos(\theta_{n,n+1}))^2 + (\sin(\bar{\theta}) - \sin(\theta_{n,n+1}))^2\right]}. \tag{S1}$$

Here $\bar{\theta}$ is the expected phase configuration (e.g., $\bar{\theta} = 0$ for in-phase configuration). At the end of each simulation we classify the formation of the expected state successful when $\text{ERR}(\bar{\theta}) \leq 0.05$. The probability is then calculated as the number of successful formations over number of realisations. In Fig. S4(b) we show the probability of in-phase (blue whole

line) and anti-phase (red dot-dashed line) configurations forming in a polygon of $N = 6$. The results evidence that non-frustrated polygons have step-like domain walls separating the regimes of in-phase and anti-phase configurations. This is in contrast to the frustrated (e.g., $N = 5$) polygons shown in Fig. S4(a) where the vortex formation probability follows a more complex distribution.

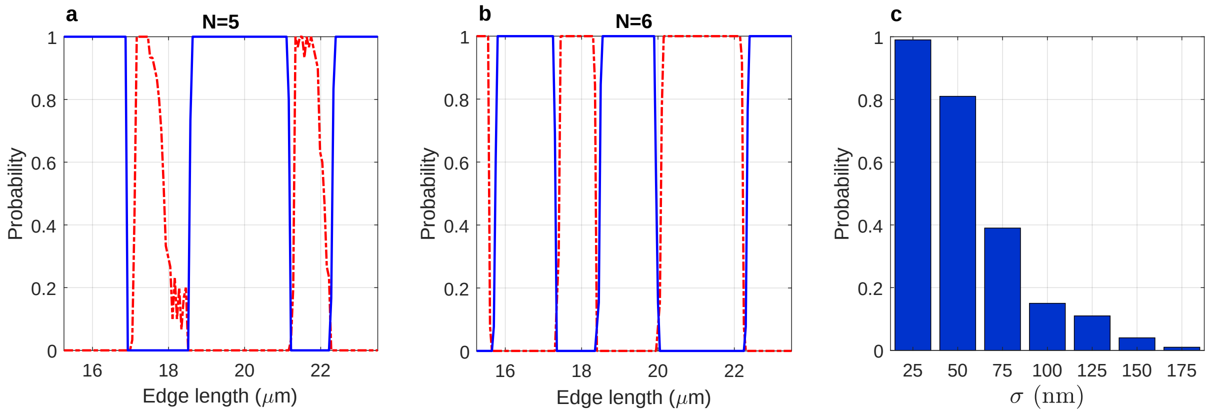


FIG. S4. **Statistical analysis on vortex state formation.** (a) Simulated probability of $|m| = 2$ vortex formation (red dot-dashed line) against probability of in-phase configuration (blue whole line) using Eq. 1 for a pentagon pumping geometry. The average is done over 30 realisations of stochastic initial conditions. The horizontal axis denotes the short distance between condensates (the edges of the polygon). (b) Simulated probability of an anti-phase formation (red dot-dashed line) against probability of in-phase configuration (blue whole line) using Eq. 1 for a hexagon pumping geometry. (c) Probability of $|m| = 2$ vortex formation for increasing standard deviation in the randomly displaced vertex coordinates of the pentagon.

We also investigate the formation probability of a $|m| = 2$ vortex state for non-ideal pentagons. We introduce uncertainties to the coordinates of each pump vertex written $(x_n + dx,\ y_n + dy)$. Here, $dx, dy$ are normally distributed random variables with zero mean and standard deviation $\sigma$. In Fig. S4(c) we show the drop in probability of the $|m| = 2$ vortex forming for increasing standard deviation. These results carry an important message. As the the number of pump spots increases the probability of maintaining a perfectly regular polygon drops since experimental uncertainties are never fully avoided, and therefore the probability of observing a vortex state diminishes. This challenge should then be overcome by either designing an alternative discrete rotational geometry which favours more strongly

the formation of vortex states, or through future design of higher quality experiments.
